# An N-Myristoylated Globin with a Redox-Sensing Function That Regulates the Defecation Cycle in *Caenorhabditis elegans*

**DOI:** 10.1371/journal.pone.0048768

**Published:** 2012-12-12

**Authors:** Lesley Tilleman, Sasha De Henau, Martje Pauwels, Nora Nagy, Isabel Pintelon, Bart P. Braeckman, Karolien De Wael, Sabine Van Doorslaer, Dirk Adriaensen, Jean-Pierre Timmermans, Luc Moens, Sylvia Dewilde

**Affiliations:** 1 Department of Biomedical Sciences, University of Antwerp, Antwerp, Belgium; 2 Department of Biology, Ghent University, Ghent, Belgium; 3 Department of Chemistry, University of Antwerp, Antwerp, Belgium; 4 Department of Physics, University of Antwerp, Antwerp, Belgium; 5 Institute of Structural Chemistry, Chemical Research Center of Hungarian Academy of Sciences, Budapest, Hungary; 6 Department of Veterinary Sciences, University of Antwerp, Antwerp, Belgium; University of South Florida College of Medicine, United States of America

## Abstract

Globins occur in all kingdoms of life where they fulfill a wide variety of functions. In the past they used to be primarily characterized as oxygen transport/storage proteins, but since the discovery of new members of the globin family like neuroglobin and cytoglobin, more diverse and complex functions have been assigned to this heterogeneous family. Here we propose a function for a membrane-bound globin of *C. elegans*, GLB-26. This globin was predicted to be myristoylated at its N-terminus, a post-translational modification only recently described in the globin family. *In vivo*, this globin is found in the membrane of the head mesodermal cell and in the tail stomato-intestinal and anal depressor muscle cells. Since GLB-26 is almost directly oxidized when exposed to oxygen, we postulate a possible function as electron transfer protein. Phenotypical studies show that GLB-26 takes part in regulating the length of the defecation cycle in *C. elegans* under oxidative stress conditions.

## Introduction

Myristoylation is a modification by which a 14-carbon fatty acid myristate is covalently attached to the N-terminal glycine residue of proteins [1]–[3]. The modification is catalyzed by the enzyme N-myristoyltransferase, and occurs most commonly on glycine residues exposed during co-translational N-terminal methionine removal [4]. Myristoylation also occurs post-translationally, for example when internal glycine residues become exposed by caspase cleavage during apoptosis [5]–[10]. N-myristoylation promotes weak and reversible protein-membrane and protein-protein interactions [11], [12], and is important for the *in vivo* localization and the role that proteins can play in e.g. signal transduction, oncogenesis and viral replication. A wide range of proteins is known to be myristoylated at the N-terminus, like the catalytic subunit of PKA cAMP-dependent protein kinase [13], calcineurin B [14], and the non-receptor tyrosine kinase c-Src [15]. Recently, acylation sites were also discovered in other classes of proteins, e.g. the globin family.

Globins occur in all kingdoms of life where they participate in a wide variety of processes, like oxygen sensing [16], NO detoxification [17], [18], and behavior [19], [20]. While globin association with the cell membrane (like *Vitreoscilla* hemoglobin) has been identified in some bacteria [21], only a few globins were known to be anchored in it through covalent attachment of fatty acids. This view changed in 2010 when the hemoglobin of the green shore crab *Carcinus maenas* was reported to possess a myristoylation site at its N-terminus, localizing it in the membrane of the gill's chief cells, where it could play a role in the protection of membrane lipids from ROS [22]. Globin X, found in fishes and amphibians, and some of the globins of the acorn worm *Saccoglossus kowalevskii*, possess a myristoylation and a palmitoylation site that are both required for correct targeting and membrane localization [23], [24].

In our search for myristoylation sites in the group of 33 globins of the nematode *Caenorhabditis elegans* (*C. elegans*) [25]–[28], we found that GLB-26, already partially characterized by our group [29], [30], was predicted to be myristoylated at its N-terminus. Recombinant GLB-26 is a globin that exhibits hexacoordination of the heme iron and was purified in the ferric low-spin state. A stable oxygenated species could not be produced since the heme iron atom, when exposed to O_2_, was almost directly oxidized, thereby reducing the bound diatomic ligand [29]. Therefore, a function as O_2_ transporter was excluded and other possible roles were suggested. Given the fast oxidation of the heme iron atom, a role in redox reactions seemed most plausible. A model of the 3D structure of GLB-26 was constructed, supporting this idea [28]. Several possible reaction mechanisms were suggested. In the case that O_2_ is reduced, the superoxide ion O_2_^−^, can participate in redox reactions or can dismutate into O_2_ and H_2_O_2_. The latter can then act as a signaling molecule *in vivo* [31]–[33]. In this case, GLB-26 could serve a role as oxidase. Localization studies where *glb-26* promotor::*gfp* fusion constructs were generated and injected into the gonads of young adult hermaphrodites [27], localized GLB-26 in the head mesodermal cell and the tail stomato-intestinal muscle cell. Since these are the sites where food intake and defecation take place, a role of GLB-26 in the defecation cycle of *C. elegans* can be emphasised. Defecation in *C. elegans* is a three-part motor program (DMP) that recurs every 45.3 (±4.3) seconds in well-fed wild type worms [34]–[36]. Three sets of muscles are activated sequentially, triggered by an endogenous ultradian clock [36], [37]. First the posterior body-wall muscles near the tail contract simultaneously, pressurizing and pushing gut contents through it (pBoc). These muscles then relax and about 2 seconds later anterior body-wall muscles simultaneously contract, forcing the pharynx back against the intestinal lumen (aBoc). Immediately following the anterior contraction, muscles that open the anus contract to release the pressurized gut contents, which is called the expulsion (exp) [35]. The study of motor program mutants and motor neuron mutants indicated that activation of the muscles, activation of the motor neurons, completion of the motor program, and release of gut pressure are not necessary for generation of the normal DMP rhythm [36]. Specific proteins, however, collaborate to regulate the defecation cycle in a positive or negative manner. These include proteins encoded by class 1 *flr*-genes, like FLR-1, an ion channel of the epithelial sodium channel/degenerin superfamily, and FLR-4, a protein kinase with a hydrophobic domain at the carboxyl terminus [37].

We proved that myristoylation plays a crucial part in targeting GLB-26 to the cell membrane. We also show that GLB-26 is present in the head mesodermal cell, the tail stomato-intestinal muscle cell, and the anal depressor muscle cell. We further suggest that this globin is involved in the defecation cycle of *C. elegans*, and that it serves a role in regulating the length of this rhythmic behavior under oxidative stress conditions.

## Materials and Methods

### Prediction of N-terminal myristoylation and subcellular localization of GLB-26

The prediction of the post-translational modification was done by the Myristoylator Prediction Program (www.expasy.org/tools). The subcellular localization of GLB-26 was predicted using the General Eukaryotic Localization Prediction Program WoLF PSORT [38], the Euk-mPLoc server version 2.0 [39]–[44], the Balanced Subcellular Localization Predictor [45], and the Subnuclear Compartments Prediction System [46], [47].

### Cloning of *glb-26*

*C. elegans* worms were grown, total RNA was isolated, and cDNA was prepared as described previously [25], [29]. The cDNA of wild type *glb-26* was amplified using the gene specific forward and reverse primers 5′– GGAAGATCTCATGGGCTCCTCTACTTCGACTCCTGC–3′ and 5′– CCCAAGCTTCTCCTCATCGTCTTCTTTTGTTTC–3′ respectively. Wild type GLB-26 contains two cysteine residues that can cause aggregation due to the formation of disulfide bridges. To avoid this, both cysteine residues were mutated to serine (GLB-26*) using the Quickchange™ site directed mutagenesis kit (Stratagene) as described earlier [48], using mutation primers 5′–GGACCGTCAAACTCTGGAAGTACAATAACG–3′, and 5′–CGTTATTGTACTTCCAGAGTTTGACGGTCC–3′ for the first cysteine, and 5′– CGAAACTTTCTCAAGAAATCGGC–3′ and 5′–GCCGATTTCTTGAGAAAGTTTCG–3′ for the second cysteine. To prove myristoylation of GLB-26*, the myristoylation site, glycine at position 2, was modified to alanine, with the mutation primers 5′– GATCTCATGGCCTCCTCTACTTCG–3′ and 5′– CGAAGTAGAGGAGGCCATGAGATCTGA–3′. This mutant is annotated as GLB-26*G2A. Both cDNAs were cloned into the pEGFP-N1 vector (Clontech) using BglII and HindIII restriction enzymes (Biolabs, Westburg), resulting in two expression constructs : pglb-26*-egfp-N1 and pglb-26*G2A-*egfp*-N1. Ligation of cDNA in the vector was performed using T4 DNA ligase (Novagen).

### *In vitro* sublocalization of GLB-26

Human neuroblastoma SH-SY5Y cells (ATCC® CRL-2266™) were cultured as per manufacturer's protocol. Cells were seeded in 10 mm cell culture dishes with glass bottom (Greiner bio-one) one day before transfection at a density of 5×10^4^ cells per cm^2^. They were transfected with 3 µl of lipofectamine™ 2000 and 0.5 µg of pEGFP-N1 plasmid containing the cDNA for *glb-26* or *glb-26G2A* according to the manufacturer protocol. After four hours, the transfection medium was replaced by growth medium and SH-SY5Y-cells were allowed to express the GFP-tagged GLB-26 proteins for 24 hours. Finally, the sublocalization was examined with an Ultra*VIEW* VoX microscope (PerkinElmer), and images were obtained with the Volocity 6.0.1 software. GFP was excitated at 488 nm with a solid state laser and a bandpass filter was used to allow emission light between 500 nm and 550 nm.

### *In vivo* localization of GLB-26

The translational reporter for *glb-26* was constructed using fusion PCR, as described by Hobert and coworkers [49]. The reporter contains 2.12 kb upstream and 0.90 kb downstream of the *glb-26* gene, to include endogenous promoter and 3′UTR elements. The *gfp* gene was amplified from the vector pPD95.75 (Fire Lab), and fused at the 3′ side of the *glb-*26 coding gene, thereby preceding the *glb-26* 3′UTR region. For *glb-26*, the primers used were the forward primer 5′–TGAAGATGGTGGTACAAAGT–3′ to amplify the promoter sequence, the forward nested primer 5′–GTAAAACTTTGGGTTGGTCT–3′ for the promoter sequence, the reverse primer 5′–AGTTCTTCTCCTTTACTCAACTCCTCATCGTCTTCTTTTG–3′ for the *glb-26* gene, the *glb-26* 3′UTR forward primer 5′– GCATGGATGAACTATACAAATGAATGTGTGATTTTTTGAT–3′, the *glb-26* 3′UTR reverse primer 5′–GAAATGTGCTCTCTATGAGG–3′, and the *glb-26* 3′UTR reverse nested primer 5′–GCACTTGTGACGTTTTCTAT–3′. For the *gfp* gene, the forward primer 5′– TTGAGTAAAGGAGAAGAAC–3′ and the reverse primer 5′– TTTGTATAGTTCATCCATGCC–3′ were used.

The *unc-119* gene, including a 2.189 kb upstream region and a 1.228 kb downstream region of the *unc-119a* isoform, was amplified with the forward primer 5′– TCAGTAAAAGAAGTAGAAT–3′ and reverse primer 5′–GAATTTTAACAATACTTC–3′. The PCR product was used as a co-injection marker, and rescued the locomotion defect of the *unc-119(ed3)* strain, i. e. the strain used for microinjection.

The final PCR products were injected into the gonads of young adult hermaphrodites using an AxioVert 135 (Zeiss) microscope and FemtoJet microinjection system (Eppendorf), at a concentration of 50 ng/µl for the *glb-26* reporter and 20 ng/µl for the *unc-119* gene. Transformed lines were analyzed using a Nikon Eclipse TE2000-5 confocal microscope.

### Electron Paramagnetic Resonance measurements

The X-band continuous-wave (CW) Electron Paramagnetic Resonance (EPR) experiments were performed on a Bruker ESP300E spectrometer (microwave (mw) frequency 9.45 GHz) equipped with a gas-flow cryogenic system, allowing operation from room temperature down to 2.5 K. The magnetic field was measured with a Bruker ER035M NMR gaussmeter. The spectra were recorded at a temperature of 10 K, a microwave power of 2 mW, a modulation amplitude of 1 mT and a modulation frequency of 100 kHz.

X-band pulsed EPR experiments were performed on a Bruker Elexsys instrument equipped with Helium cryostat (Oxford Inc.). The measurements were done at 7 K. The HYSCORE (hyperfine sublevel correlation) spectrum [50] was recorded using the pulse sequence: π/2-*τ*-π/2-*t*_1_-π-*t*_2_-π/2-*τ*-echo, with *t*_π/2_ = 16 ns and *t_π_* = 32 ns; *t*_1_ and *t*_2_ were varied in step of 16 ns (matrix dimension [350×350]). Spectra were recorded for *τ* = 96, 120 and 192 ns. A four-step phase cycle was performed in all cases to remove the unwanted echoes. The HYSCORE traces were baseline corrected using a third-order polynomial, apodized with a Hamming window and zero-filled. After Fourier transformation the absolute-value spectra were computed and the spectra recorded at different τ-values were added together.

All EPR simulations were done with the EasySpin program, a Matlab toolbox developed for EPR simulations [51]. The HYSCORE spectra were simulated assuming two-spin *S* = 1/2, *I* = 1 systems using the parameters in Table 1. The simulated spectral contributions for the heme and His nitrogens were then added together. Simulations were performed of the different experimental τ-values.

**Table 1:** Nitrogen hyperfine and nuclear quadrupole components derived from the simulations for GLB-26 in comparison to other globins.

		Heme ^14^N nuclei		His ^14^N nuclei		
	*g*_max_	|A_zz_|/MHz	|Q_zz_|/MHz	|A_zz_|/MHz	|Q_zz_|/MHz	Ref.
GLB-26	3.25 (±0.01)	5.65 (±0.20)	0.42 (±0.05)	4.65 (±0.10)	0.90 (±0.10)	This work
CYGB	3.20	5.45	0.42	5.00	0.90	[68]
NGB ΔCys	3.10	5.70	0.43	4.90	0.85	[66]
*Gs*GCS^162^	2.925	5.40	0.46	5.90	0.75	[67]

### Reduction potential

#### Chemicals and solutions

Mercaptohexanol (MOH), 2-[4-(2-hydroxyethyl)-piperazinyl]ethanesulfonic acid (HEPES) and sodium hydroxide were purchased from Sigma-Aldrich. The HEPES buffer solution of 10 mmol L^−1^ was set to pH 7.0 using a 0.15 mol L^−1^ NaOH solution. Type B gelatin (Gel, IEP = 5, Bloom strength = 257), isolated from bovine skin by the alkaline process, was kindly supplied by Tessenderlo Chemie (Belgium).

#### Electrode preparation

The three-electrode system consists of a saturated calomel reference electrode (SCE, Radiometer Analytical, France), a graphite counter electrode and a gold inlaid disc electrode. The gold working electrodes of 1.6 mm diameter were pretreated by mechanical polishing and modification. Before its first use, the electrode surface was briefly polished on an aluminum oxide film disc of small particles to obtain a smooth and clean surface. To remove any adherent Al_2_O_3_ particles the electrode surface was rinsed thoroughly with deionised water in an ultrasonic bath and dried with a tissue. Secondly, the gold electrode was modified by immobilization of MOH by hanging the bare electrode for 12 hours in a 14 mmol L^−1^ MOH solution. This electrode is denoted as MOH|Au.

To immobilize a drop dried layer of gelatin B onto a MOH|Au electrode, 10 µL of a gelatin B solution (5 w%)/HEPES mixture was placed on the surface by means of a pipette and was left to dry to air at 4°C. The gelatin B solution was prepared by mixing the gelatin B powder and the HEPES buffer solution at approximately 40°C, as was described earlier [52], [53] These electrodes are referred to in the text as GelB|MOH|Au. If a GLB solution is incorporated in the gelatin matrix, the final electrodes are denoted as GLB-26|GelB|MOH|Au (3∶7 ratio). The concentration of gelatin is 5 m%.

#### Apparatus

A μ-Autolab potentiostat controlled by GPES 4.9 007 software package (Metrohm, The Netherlands) was used for recording the cyclic voltametric curves. The solutions were thoroughly deoxygenated by bubbling with nitrogen for at least 30 minutes, and again for approximately 5 minutes in the cell itself before usage.

### Functional characterization of GLB-26

#### *C. elegans* strains

The *C. elegans* strains Bristol N2 (wild type strain) and *unc-119(ed3)* were obtained from the *Caenorhabditis* Genetics Center (CGC, University of Minnesota). The *glb-26 (tm4837)* knockout strain was provided by the Japanese National Bioresource Project. *glb-26 (tm4837)* was outcrossed eight times with N2 to remove adventitious mutations. Primers used to genotype *glb-26 (tm4837)* were 5′ - AGCGGGCTCGATACGAATAA – 3′ and 5′ – CACATACTGCTCGAACTGCA – 3′. Strains were cultured and experiments were carried out at 20°C on nutrient agar plates seeded with *Escherichia coli* OP50.

#### Paraquat sensitivity

For paraquat (1,1′-dimethyl-4,4′-bipyridinium dichloride, PQ) sensitivity assays, worms were synchronized by hypochlorite treatment, and isolated eggs were allowed to hatch at 20°C overnight. Hatched larvae were grown to 1- day-old worms, after which they were transferred to nutrient agar plates seeded with a spot of *E. coli* OP50, and with a final concentration of 10 mM paraquat. This concentration was used since at lower concentrations no effect on the defecation cycle was observed. At higher concentrations worms were severely affected, showing almost no movement, no pharyngeal pumping and no defecation cycle. The strongest response was found at 10 mM paraquat, and was therefore chosen in this study. Survival was monitored every 24 hours. Worms that failed to respond to repeated touching with a platinum wire were considered to be dead. PQ sensitivity assays were performed in triplicate, with approximately 200 worms per strain per trial. The values for the individual time points of the three replicates were averaged, and were used to generate survival curves. Student's t-test was used for statistical analysis.

#### Defecation cycle length

One-day-old adult hermaphrodites were obtained as described in the PQ sensitivity assay, and were used to measure the defecation cycle length as the average time between consecutive pBoc contractions. Adults were picked to a fresh nutrient agar plate seeded with a spot of *E. coli* OP50, after which they were allowed to settle down for at least 10 minutes. In the assays where PQ was included, a final concentration of 10 mM PQ was added to the plate 3 h before worms were transferred. Individual worms were followed for approximately 5 minutes, after which the average defecation cycle length was calculated. Results represent 3 independent trials with at least 5 worms per trial. Student's t-test was used for statistical analysis.

## Results

### *In silico* search for N-myristoylation and subcellular localization of GLB-26

**Figure 1:**
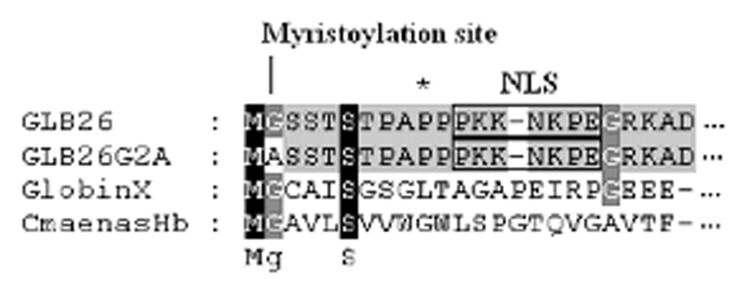
Alignment of the myristoylated globins GLB-26, GLB-26G2A, Globin X, and *Cmaenas*Hb. The myristoylation sites of all globins, and the predicted NLS of GLB-26 are indicated.

Using the Expasy Myristoylator tool [54], GLB-26 was predicted to be myristoylated on the glycine residue at position 2 with a probability of 99% (Figure 1). Myristoylation is assumed to target GLB-26 to the membrane of a subcellular compartment. To predict which compartment, we launched its primary sequence in different software programs. With the WoLF PSORT software [38] the classical nuclear localization signal PKKNKPE was found starting at position 12, and a probability of 59% that GLB-26 is transported to the nucleus *in vivo* was predicted. This was confirmed by other prediction programs. The Subnuclear Compartments Prediction System was run to predict in which part of the nucleus GLB-26 is localized, yielding the presence of GLB-26 in the nuclear lamina [46], [47].

### *In vitro* subcellular localization of GLB-26*

**Figure 2:**
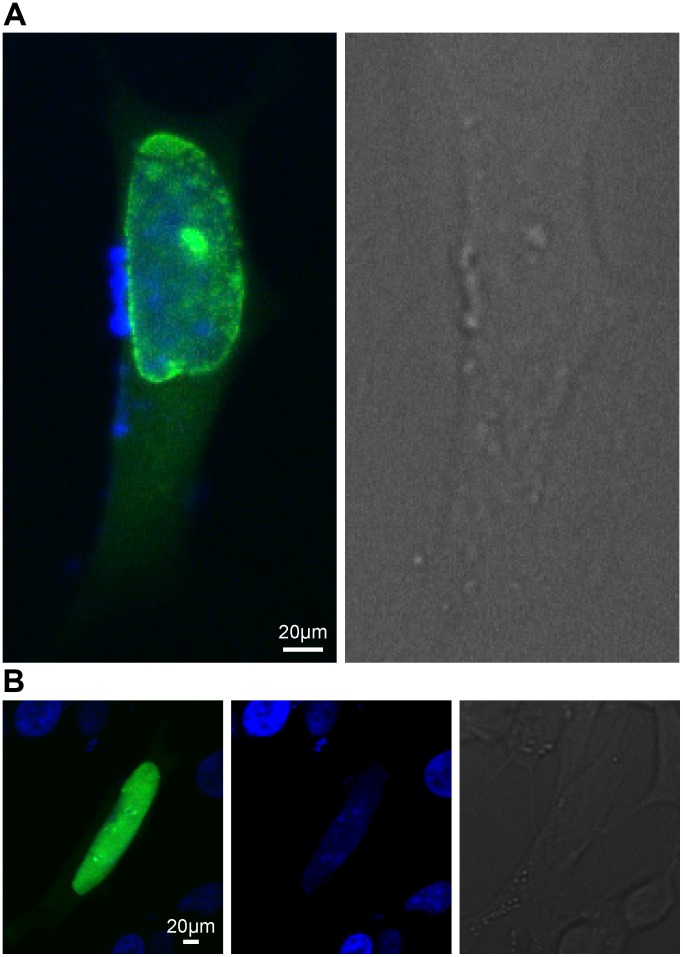
*In vitro* localization of GLB-26. Neuroblastoma SH-SY5Y cells were transfected with A - 0.5 µg of the pglb-26*- *egfp*-N1 plasmid, and B – 0.5 µg of the pglb-26*G2A- *egfp*-N1 plasmid. GLB-26* was expressed in the nuclear lamina, GLB-26*G2A was localized inside the nucleus (green fluorescence). The nucleus was visualized with Dapi staining (blue fluorescence), and presented in a separate image for clarity.

Expression of the pglb-26* - egfp - N1 plasmid in neuroblastoma cells (SH-SY5Y) showed that GLB-26* is predominantly localized in the lamina of the nucleus, as predicted by the software programs used (Figure 2A). Occasionally GLB-26* was also observed in the cellular membrane, although expression in the nuclear lamina was more prevalent (data not shown). Deletion of the myristoylation site in the glb-26* gene by site directed mutagenesis resulted in the expression of GLB-26*G2A in the nucleoplasm of the cells (Figure 2B), proving that GLB-26* is transported into the nucleus and that myristoylation of the N-terminal glycine residue is responsible for the insertion of the wild-type protein into the nuclear membrane.

### *In vivo* localization of GLB-26

**Figure 3:**
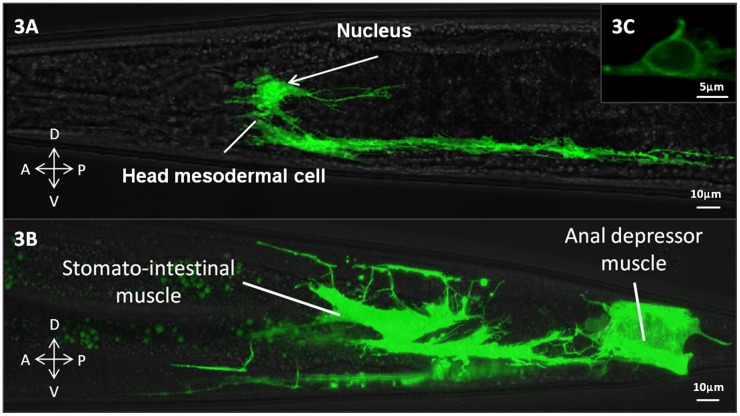
*In vivo* expression pattern of the GLB-26 translational reporter. A) In the head region expression is seen in the head mesodormal cell, with apparent enrichment in the nucleus, B) In the tail region expression is present in the stomato-intestinal and anal depressor muscle cells, C) Detailed analysis shows that GLB-26 is membrane bound. Arrows indicate the orientation of the cells with A : Anterior side, D : Dorsal side, P : Posterior side, V : Ventral side.

A translational reporter for GLB-26 was created to determine the gene product expression pattern in *C. elegans*. This reporter includes the endogenous promoter, introns and 3′UTR, and has GFP fused to the C-terminus of GLB-26. Confocal images showed that GLB-26 is expressed in the head mesodermal cell (Figure 3A), and in the tail in the stomato-intestinal and anal depressor muscle cell (Figure 3B). GLB-26 was also found to be expressed in the male anal depressor muscle (not shown). The expression pattern observed here is in accordance with the results of a localization study conducted by Hoogewijs and coworkers, who used a transcriptional reporter [27]. Finally, this reporter also shows that GLB-26 is membrane bound (Figure 3C).

### EPR measurements

**Figure 4:**
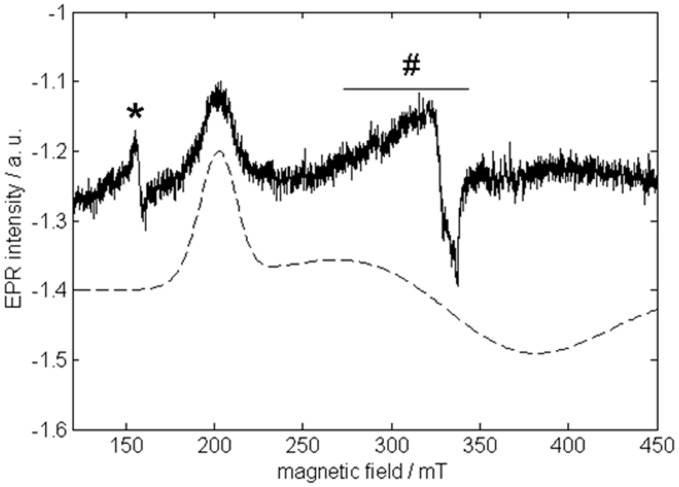
CW-EPR spectrum of ferric GLB-26. Top: Experimental X-band CW-EPR spectrum of ferric GLB-26 in a phosphate buffer (pH7). The peaks indicated by * are due to extra heme iron. # indicates a Cu(II) background signal from the cavity. The spectrum was recorded at a temperature of 10 K. Bottom: Simulation of the dominant low-spin ferric heme contribution.

**Figure 5:**
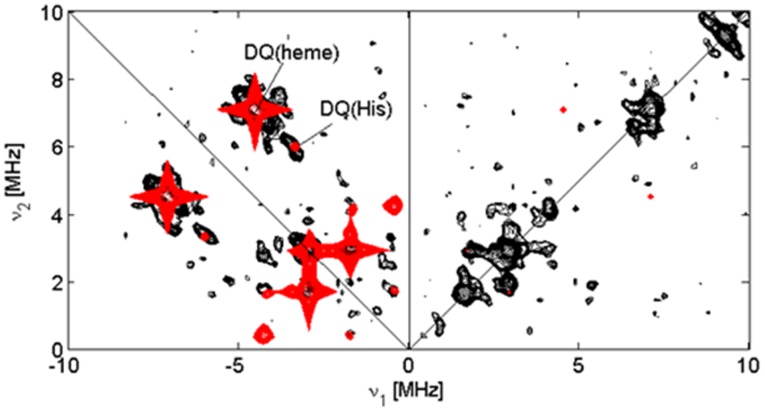
Experimental (black) and simulated (red) HYSCORE spectra taken at the magnetic field position corresponding with *g* = *g*_max_. The double-quantum (DQ) cross peaks of the heme ^14^N and His ^14^N nuclear frequencies are indicated. Both simulated and experimental spectra are sums of the spectra with τ = 96, 120 and 192 ns.

The CW-EPR spectrum of as-purified (ferric) GLB-26 (Fig. 4) is dominated by a signal stemming from a low-spin (*S* = 1/2) ferric heme. In the low-field part of the spectrum (not shown) a minor contribution stemming from a high-spin (*S* = 5/2) ferric heme is found. The latter can be assigned to a small (partially) denatured portion of GLB-26, as observed for example in cytochrome c-554 from *Nitrosomonas europaea* [55]. Only the low-field feature (corresponding to g = 3.25 (±0.01)) of the low-spin contribution can be clearly discerned. This observation, combined with the large value of *g* (considerably higher than 3) is typical for so-called ‘large *g*_max_’ or type-I EPR signals, also referred to as the highly anisotropic low-spin (HALS)-type signals [56]. Such EPR signals have been reported previously for bis-ligated ferric porphyrins and ferric cytochromes [57]–[62]. To our knowledge, GLB-26 is a rare case of a globin with a type-I (HALS) EPR spectrum. Although the *g_max_* value of ferric human neuroglobin (NGB) and human cytoglobin (CYGB) is also significantly larger than 3 [63], the EPR features are still more typical of a normal rhombic or type-II EPR signal. One of the three ferric heme forms found for hemoglobin 1 of *Drosophila melanogaster* (*Dm*Hb1) is characterized by a HALS-type EPR spectrum with even larger *g*_max_ (3.50), but little is known about the specific ligation of the heme in this form or about the function of this form [64]. In the case of GLB-26, a bis-histidine coordination of the heme group (binding of E7His and F8His) is assumed [29]. For bis-imidazole-coordinated ferric porphyrins, large *g*_max_ signals are indicative of dihedral angles between the imidazole planes larger than ∼60° [65]. Detailed information on the orientation of the imidazole planes relative to each other and to the heme can be obtained from a pulsed EPR analysis [64], [66]. However, since only the low-field EPR signal is clearly resolved in the case of GLB-26 (Fig. 4), relevant pulsed EPR spectra could only be obtained at this field position. Figure 5 shows the HYSCORE spectrum and its simulation for this observer position. The HYSCORE spectrum reflects the nuclear frequencies stemming from the interaction of the unpaired electron with the ^14^N nuclei of the heme and His ligands. Table 1 gives the nitrogen hyperfine and nuclear quadrupole components derived from the simulations for GLB-26 in comparison to other globins. While the parameters of the heme nitrogens of GLB-26 are similar to those of other globins, there is quite a strong variation in the hyperfine values stemming from the Fe-coordinating His nitrogens for the different globins indicative of the marked difference in the imidazole orientation and N_His_-Fe binding strength (Table 1). The A_zz_ value of the His ^14^N of GLB-26 not only differs strongly from that of the globin domain of the globin-coupled sensor of *Geobacter sulfurreducens*, a globin in which the two axial heme-ligating His groups have almost coplanar imidazole planes [67], it is also smaller than for the NGB and CYGB cases [66], [68].

### Redox chemistry

**Figure 6:**
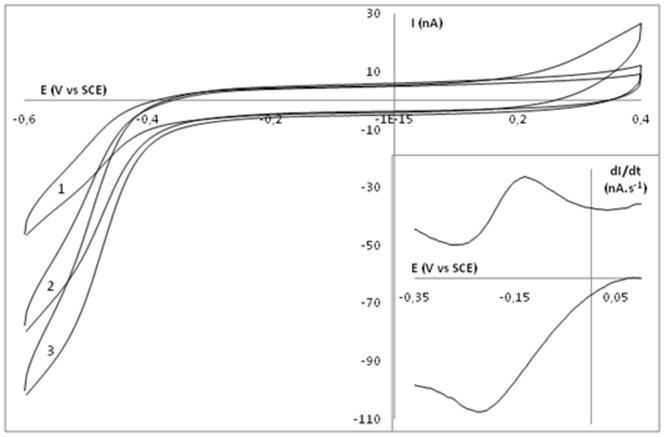
The current-potential behavior of a GLB-26|GelB|MOH|Au electrode with different GLB-26 concentrations (mmolL^−1^): 0.1 (1), 0.2 (2) and 0.4 (3) in a 10 mmol L^−1^ HEPES pH 7 buffer solution with a scan rate of 50 mVs^−1^. Inset: Derivative of the current-potential behavior of a GLB-26|GelB|MOH|Au electrode in the presence of hydrogen peroxide.

To unravel the redox potential of GLB-26 and its function, we encapsulated the protein in a biocompatible gelatin matrix on top of a MOH modified gold electrode. Figure 6 represents the current-potential behavior of a GLB-26|GelB|MOH|Au electrode with different GLB-26 concentrations (curve 1–3) in a 10 mmol L^−1^ HEPES buffer solution in a potential window from −0.6 to 0.4 V with a scan rate of 50 mVs^−1^. Gelatin is not electrochemically active in this potential range [69]. The redox process at ca. −0.47 V vs SCE (−0.27 V vs SHE), observed in curve 1–3, can be explained as the reaction of one of the heme groups (Fe^3+^/Fe^2+^) present in the globin protein, since no other redox active elements are present in the protein. When the GLB-26 concentration increases, the redox current increases as well, following the Randles-Sevcik equation [70], [71]. It is shown that after adding hydrogen peroxide, no electrocatalytic wave is observed at this redox potential, indicating that this low-potential heme site doesn't show electrocatalytic properties towards hydrogen peroxide. Since the applied potential is highly negative, stressing the native conformation of GLB-26, this redox couple might be due to a (partially) denatured fraction of the protein sample. This is consistent with the minor high-spin fraction of GLB-26 observed by CW-EPR measurements. However, at a higher potential, a sigmoidal wave with a limiting current appears, after addition of hydrogen peroxide, corresponding to an enzymatic velocity, and this wave is centered at a potential E_cat_. As given in the inset of Figure 6, the peaks are centered at an E_cat_ of ca. −0.17 V vs SCE (+0.03 V vs the standard hydrogen electrode, SHE). Therefore, this high-potential heme group functions as the electron relay site. To investigate the possible formation of a ferryl-oxo group by the subsequent reaction of ferric GLB-26 with hydrogen peroxide, a process often observed in peroxidases [72], H_2_O_2_ was added in equimolar proportion to ferric GLB-26, and the reaction was followed spectroscopically. No change in absorbance was measured confirming the absence of the formation of a ferryl intermediate (not shown).

### Phenotypical characterization of the *glb-26* knock-out strain

**Figure 7:**
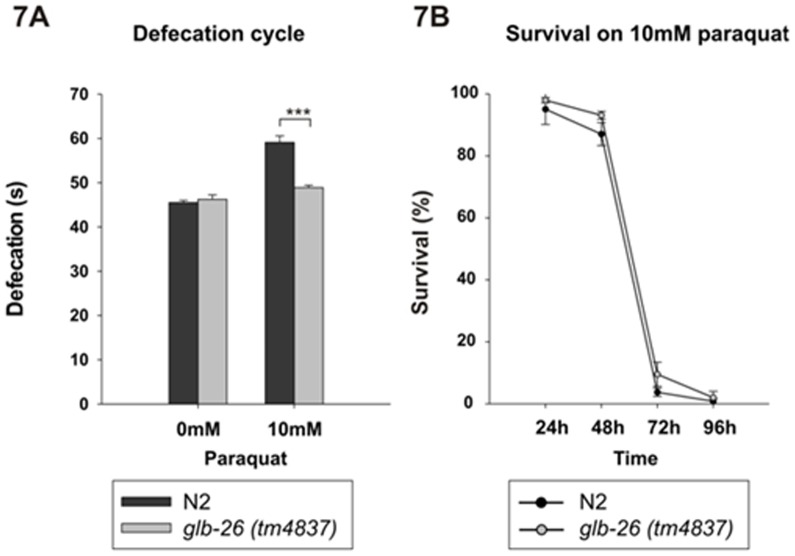
Influence of PQ on wild type N2 and *glb-26(tm4837)*. **A**) Defecation cycle under normal conditions does not significantly differ between the wild type N2 and *glb-26(tm4837)*. When exposed to 10 mM PQ, the wild type shows a strong slowing down of the defecation cycle, whereas this slowing down is weaker in *glb-26(tm4837)*. Error bars indicate standard error (n = 3), * p<0.05; *** p<0.001. **B**) Survival of N2 and *glb-26 (tm4837)* on 10 mM PQ does not significantly differ. Error bars indicate standard error (n = 3).

The *glb-26 (tm4837)* strain is homozygous viable and has no obvious phenotypic defects. Because GLB-26 is expressed in muscles involved in the defecation cycle of *C. elegans*, we compared the defecation rates of the *glb-26(tm4837)* and the wild type strain. The *glb-26 (tm4837)* mutation removes 221 of the 552 coding nucleotides of GLB-26 and is likely a null mutation. No significant difference in defecation rate was seen under normal conditions between the wild type and *glb-26 (tm4837)* strain, indicating that GLB-26 is not necessary for the control of the defecation cycle under normal conditions. Geuens and coworkers showed that GLB-26 cannot function as an oxygen carrier, and instead suggested that it might function in redox reactions/signaling [29]. Therefore, the defecation cycle was analyzed in worms exposed to PQ, an environmental toxin that induces oxidative stress. *In vivo*, PQ will become part of redox cycling, leading to the generation of superoxide on one hand, and to the oxidation of reducing equivalents (e.g., NADPH, reduced glutathione) on the other hand [73]. This makes it a good compound to study any form of redox signaling or detoxification of reactive compounds. Interestingly, exposure of the worms to 10 mM PQ significantly (p = 3E-9) slowed down the defecation cycle in the wild type strain, while in the *glb-26 (tm4837)* strain only limited slowing down was seen (p = 0.03) (Fig. 7A). GLB-26 thus seems to be involved in the defecation cycle when worms are exposed to conditions that alter the redox status. To test if GLB-26 could specifically be involved in detoxification of reactive compounds, the survival rate of worms continuously exposed to 10 mM PQ was analyzed (Figure 7B). After 24 h, the first negative effects on survival could be seen, and after 72 h, the majority of worms had died. In this case, however, no significant difference was seen between the wild type and *glb-26 (tm4837)*, although the latter appeared to survive slightly better than the wild type.

## Discussion

**Figure 8:**
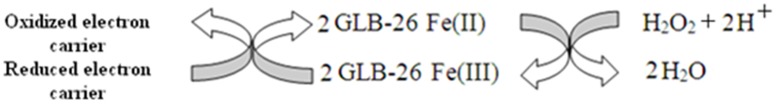
Reduction cycle of GLB-26. GLB-26 can transfer an electron to H_2_O_2_, and can in turn be reduced by a yet unknown electron carrier. This electron shuttle results in a prolonged defecation cycle in *C. elegans*.

Given the fast autoxidation and strong hexacoordination of GLB-26, this globin has previously been suggested to have a redox function *in vivo* [25]–[30]. This was confirmed by EPR and electrochemical experiments performed in this work. Indeed, the ^14^N-His hyperfine parameters of GLB-26 vary from those of other bis-His-ligated globins (Fig. 5, Table 1) and the CW-EPR spectrum of ferric GLB-26 (Fig. 4) resembles those reported for bis-histidine-coordinated cytochromes [59], [62], indicating dihedral angles between the imidazole planes of GLB-26 of 55-60°. Moreover, it was shown that homodimeric GLB-26 possesses two low-spin, high potential heme sites (Fig. 6) comparable to the high potential heme site of cytochrome bc_1_ of the purple bacteria *Blastochloris* [74], and cytochrome c peroxidase of *Paracoccus denitrificans* [75]. The two high potential heme sites of GLB-26 show electrocatalytic activity towards H_2_O_2_, a type of ROS (Fig. 8). moreover, earlier work showed that GLB-26 can transfer an electron to cytochrome c at the same rate as mitochondrial redox carriers *in vitro* [30]. Therefore a function as a member of an electron transport chain, involving H_2_O_2_ is feasible.

To identify which process(es) GLB-26 might be involved in, the *in vivo* expression pattern was determined. GLB-26 is mainly expressed in the cellular and nuclear membrane of the head mesodermal cell and in the tail stomato-intestinal and anal depressor muscle cells (Fig. 3). The function of the head mesodermal cell is unknown, but some ideas have been postulated based on its connections with neighboring cells [77]. The head mesodermal cell has several processes on the dorsal and ventral side of the body wall. The ventral posterior arm runs in conjunction with ventral body wall muscle arms and the hypodermal ridge and makes gap junctions with ventral body wall muscle arms. The dorsal posterior process runs some distance adjacent to the dorsal hypodermal ridge and makes gap junctions with arms from dorsal muscles. The head mesodermal cell thus stays in close contact with the body wall muscles and during defecation, these body wall muscles contribute to the control of internal pressure and concentration of the gut contents before the expulsion of the waste material.

Stomato-intestinal muscles are two sheet-like cells that connect the surfaces of the intestinal cells to the ventral body wall. Contraction of these muscles promotes defecation by pressurizing the intestinal contents near the posterior end of the intestine. The anal depressor muscle is a sexual dimorphic muscle. In hermaphrodites it is involved in the defecation cycle, while in males it is specialized to function as an auxiliary spicule muscle (www.wormatlas.org).

Given the specific localization pattern of GLB-26 in sister cells of the gut, associated with defecation, and the aforementioned reactivity towards H_2_O_2_, a role for GLB-26 in the defecation cycle under oxidative stress conditions was suggested. Indeed, after addition of 10 mM PQ, a source of ROS, the defecation cycle in the wild type *C. elegans* strain was prolonged, while in the *glb-26* knock out strain this prolongation was far less pronounced, suggesting a role for GLB-26 in the regulation of the periodicity of this cycle (Fig. 7A). The addition of 10 mM PQ had no significantly different effect on the lifespan of both the *glb-26* knock-out strain and the wild type strain, indicating that GLB-12 is not involved in general protection against oxidative stress (Fig. 7B).

Other genes affecting the periodicity of the defecation cycle of *C. elegans* have been identified in the past and generally act in the gut by altering some of its properties [78]–[82]. To date, only one gene, *dsc-1*, has been identified that acts in the enteric muscle cells, i.e. the stomato-intestinal muscles and anal depressor muscle cell, through a feedback mechanism to the gut. *dsc-1* encodes a Paired-like homeodomain protein, a class of transcription factors previously associated with the terminal differentiation of neurons in *C. elegans* [82]. One type of feedback mechanism works through gut expansion and may involve a humoral signal that acts to coordinate the Ca^2+^ signal generated in the gut with the timed contractions of the various muscles types, thereby regulating the length of the defecation cycle [82].

At present, it is unclear if a similar feedback mechanism is utilized by GLB-26 and that it as such works as a signaling molecule, or its role is purely enzymatic, or both. An enzymatic function is supported by the fact that relative quantification of the *in vivo* expression levels of all globin genes of *C. elegans* showed that *glb-26* was expressed about 40 fold higher than the other globin genes under normal conditions [26], [83].

At this point we cannot assign the exact role of GLB-26 in this process however, but some suggestions can be postulated based on its reactivity towards H_2_O_2_ and the physiological significance of this ROS generation.

Several sources of ROS have been identified and one of them involves both the nematode's immune defense and the defecation cycle. *C. elegans* feeds on bacteria, but when pathogens are ingested, the innate immune defense is initiated through germ-line encoded pattern-recognition receptors to restrict the damage and to ensure its survival [84]. This response is pathogen-dependent [85] and includes the excretion of ROS in the intestinal lumen of the nematode [86], [87]. The dual oxidase Ce-Duox-1/BLI-3 was shown to play a role in the defense mechanism against pathogens as it possesses an NADPH oxidase domain to generate H_2_O_2_, an EF hand domain for enzyme activation by the binding of Ca^2+^ [88], and an extracellular peroxidase domain, that might be important for the binding of H_2_O_2_ [89], [90]. However, the role of the latter domain is still unknown [91].

In addition to the protection against pathogens, ROS increase also stimulates inositol trisphosphate (IP_3_)-mediated Ca^2+^ mobilization by increasing cytosolic Ca^2+^ accumulation through the endoplasmic reticulum, and by stimulating Ca^2+^ influx through Ca^2+^ channels. Two types of Ca^2+^ binding proteins include the IP_3_ receptor (ITR-1), and calreticulin (CTR-1), a molecular chaperone, that are expressed predominantly in the intestine of *C. elegans*. *Ctr-1* genetically interacts with *itr-1*, and shows a synergic effect on the length of the defecation cycle [76], [92], [93]. An elongation of the defecation cycle of *C. elegans* was also observed upon pathogenic ingestion [94], possibly through the same Ca^2+^ signaling pathways, and this could play an important role in microbial infections.

GLB-26 is expressed in the cellular and nuclear membrane as a result of N-myristoylation, and is therefore in close proximity of the membrane bound Ce-Duox-1/BLI-3 (Fig. 2A), that was suggested to act downstream of IP_3_ [95]. As such, GLB-26 could act as an electron donor for H_2_O_2_ and signal the presence of this reactive compound by oxidizing another electron carrier. Its dual localization in the cellular and nuclear membrane might be crucial for the fast propagation of the detection in the change of oxidative stress conditions (Fig. 7A), and to elucidate an appropriate response in the nucleus, possibly involving the modulation of transcription factors. Future experiments to identify interaction partners are necessary and will hopefully gain insight in the complex regulation of this rhythmic behavior.

## Conclusions

GLB-26 is a hexacoordinated globin that is attached to the cellular and nuclear membrane by myristoylation of its N-terminus. GLB-26 may act as a heme peroxidase, signaling the presence of H_2_O_2_ in the nucleus and in the cytosol of the head mesodermal cell, stomato-intestinal muscle, and anal depressor muscle cells. When oxidative stress conditions are induced, the redox status of the cytoplasm and hence of the nucleus is altered. This is sensed by GLB-26, and is translated in a prolonged defecation cycle of *C. elegans*. The dual expression of GLB-26 in the cytosol and in the nucleus may contribute to the fast translation of the changed redox status in the cell.
